# An Experimental Test of Buffer Utility as a Technique for Managing Pool-Breeding Amphibians

**DOI:** 10.1371/journal.pone.0133642

**Published:** 2015-07-21

**Authors:** Jessica S. Veysey Powell, Kimberly J. Babbitt

**Affiliations:** Department of Natural Resources and the Environment, University of New Hampshire, Durham, New Hampshire, United States of America; Universität Zurich, SWITZERLAND

## Abstract

Vegetated buffers are used extensively to manage wetland-dependent wildlife. Despite widespread application, buffer utility has not been experimentally validated for most species. To address this gap, we conducted a six-year, landscape-scale experiment, testing how buffers of different widths affect the demographic structure of two amphibian species at 11 ephemeral pools in a working forest of the northeastern U.S. We randomly assigned each pool to one of three treatments (i.e., reference, 100m buffer, 30m buffer) and clearcut to create buffers. We captured all spotted salamanders and wood frogs breeding in each pool and examined the impacts of treatment and hydroperiod on breeding-population abundance, sex ratio, and recapture rate. The negative effects of clearcutting tended to increase as forest-buffer width decreased and be strongest for salamanders and when other stressors were present (e.g., at short-hydroperiod pools). Recapture rates were reduced in the 30m, but not 100m, treatment. Throughout the experiment for frogs, and during the first year post-cut for salamanders, the predicted mean proportion of recaptured adults in the 30m treatment was only 62% and 40%, respectively, of that in the reference treatment. Frog sex ratio and abundance did not differ across treatments, but salamander sex ratios were increasingly male-biased in both cut treatments. By the final year, there were on average, only about 40% and 65% as many females predicted in the 100m and 30m treatments, respectively, compared to the first year. Breeding salamanders at short-hydroperiod pools were about 10% as abundant in the 100m versus reference treatment. Our study demonstrates that buffers partially mitigate the impacts of habitat disturbance on wetland-dependent amphibians, but buffer width and hydroperiod critically mediate that process. We provide the first experimental evidence showing that 30-m-wide buffers may be insufficient for maintaining resilient breeding populations of pool-dependent amphibians, at least during the first six years post-disturbance.

## Introduction

Vegetated buffers have been used extensively for several decades to protect wetlands across a variety of landscapes. Buffers were originally designed to filter water pollutants and maintain water quality [1, 2]. In this context, 15–30 m-wide buffers are often sufficient to remove nitrogen, phosphorus, and sediment from runoff before it enters wetlands [3–5]. Over the last two decades policy-makers have increasingly relied on buffers to conserve wetland-dependent wildlife [1–3]. Lacking wildlife-specific data, they assumed 15–30 m-wide water quality buffers would benefit wildlife [3, 6, 7]. Such narrow buffers may be insufficient for maintaining viable populations of many wetland-dependent species, however, because these species regularly use habitat that extends farther from wetlands than typical water-quality buffers [8–10].

This may be especially true for amphibians that breed in ephemeral pools. The semi-annual drying cycle of ephemeral pools prevents establishment of predatory fish populations, making these pools extremely productive amphibian habitat. During the non-breeding season, these amphibians range across the surrounding landscape, using additional wetlands and uplands for foraging, shelter, estivation, and hibernation [11–13], sometimes migrating hundreds of meters into terrestrial habitat [14–16]. Land-uses that alter the habitat quality of breeding pools and/or adjacent uplands can potentially have strong negative effects on local and regional population persistence.

Historically, ephemeral pools received little policy protection in the United States (U.S.). Federal protection ostensibly falls under the Clean Water Act, but is tenuous given recent Supreme Court decisions and has never officially included a buffer [17]. Some states, counties, and municipalities supplement federal law by implementing more stringent local policies. However, only 15 of 50 states have substantial wetland programs, most of which do not include protection, let alone buffers, for ephemeral pools [18]. Four of six New England states provide a regulatory buffer for ephemeral pools (mean ± SD: 23 ± 29 m; range: 0–76 m), but these buffers typically only apply to a subset of pools and project types [19–22] and are substantially narrower than the 164-290-m buffers that scientists recommend, based on amphibian migration data (e.g., [23]).

Previous observational and modeling studies conclude that regulatory buffers in the U.S. are inadequate for protecting populations of ephemeral-pool-breeding amphibians [10, 23, 24]. In most of these studies, however, buffer effects were never explicitly tested, but rather estimated from species’ migration distances in undisturbed habitat. Thus, very little is actually known about how the demographics and behavior of ephemeral-pool-breeding amphibians differ in unbuffered and buffered systems, and what optimal buffer widths are for different species. Overall, experimental confirmation of the need for wider buffers is severely lacking. Though some policy-makers express interest in expanding buffers to accommodate wildlife habitat needs [25–27], they hesitate to support policy changes without solid, experimental evidence demonstrating the utility of buffers for wildlife [28, 29].

To address this need, we conducted a six-year, landscape-scale experiment and examined how buffers of different widths affect breeding-adult demography for two amphibian species at ephemeral pools in an industrial forest of the northeastern U.S. We studied spotted salamanders (*Ambystoma maculatum*) and wood frogs (*Lithobates sylvaticus*) because they use similar macrohabitat, but differ in microhabitat preferences and key demographic traits, and may thus require different conservation strategies. In particular, spotted salamanders utilize small-mammal burrows extensively as refugia [13, 30], can live up to 32 years in the wild (mean adult age in one northern population was 8.8 years; [31, 32]), and usually breed multiple times in their lives [33]. By contrast, wood frogs seek refuge in other wetlands during the summer [11], overwinter in leaf litter [11, 34], have a maximum lifespan of 5–6 years [35, 36], and typically breed only once after reaching sexual maturity [35, 37], but are more fecund than spotted salamanders [10].

For each species, we examined whether the abundance, proportion recaptured, and sex ratio of breeding adults differed across buffer treatments and/or varied with time as disturbed forest around experimental buffers regenerated. We hypothesized that:

1. For both species, breeding-adult abundance and recapture proportion would be positively correlated with buffer width. We expect this because wider buffers provide a greater area of high-quality habitat in proximity to breeding pools and should support a larger, more stable breeding population than pools with narrower buffers.
2. For both species, breeding-adult sex ratio (i.e., the proportion of males in the breeding population) would vary with buffer width. Males of both species cluster closer to breeding pools than do females, such that generally, buffers should provide more habitat for males, and females should be disproportionately impacted by disturbances beyond the buffer [38–40]. Due to a lack of fine-grain resolution in existing knowledge of the spatial distribution of both sexes around pools, however, we did not predict directionality in the relationship between sex ratio and buffer width.
3. For both species, any negative impacts to breeding-adult abundance, recapture proportion, or sex ratio in the buffered treatments would recover with time (i.e., be restored to values similar to those in the reference treatment, after deviating from reference-treatment values at some prior time), as disturbed forests regenerated.
4. Wood frogs, being more fecund, would recover faster than spotted salamanders.

## Methods

### Study site

We conducted this research in an industrial forest, privately owned by International Paper/ Sustainable Forest Technologies, in east-central Maine, U.S. (45°0’52”N, 44°48”32”N; 68°28’11”W, 67°53’10”W). The forest is predominantly eastern hemlock (*Tsuga canadensis*) and northern hardwood *(Fagus grandifolia*, *Acer saccharum*, *Betula alleghaniensis*) at lower elevations, and balsam fir (*Abies balsamea*) and red spruce (*Picea rubens*) at higher elevations. Moderate hills, wetlands (including numerous ephemeral pools), and dirt logging roads are common. In 2002, we identified 300 ephemeral pools in this landscape and chose 40 of similar size (i.e., 0.1–0.3 ha) and adjacent forest (i.e., uncut within 1000 m) for in-depth inspection. In spring 2003, we surveyed egg masses at these 40 pools and identified 35 with breeding populations of both wood frogs and spotted salamanders and hydroperiods of at least five months post-ice out. We randomly chose 12 of the 35 pools for this study. In spring 2004, we learned that one of the 12 pools had a permanent inflow and removed that pool from the study.

### Buffer creation

**Fig 1:**
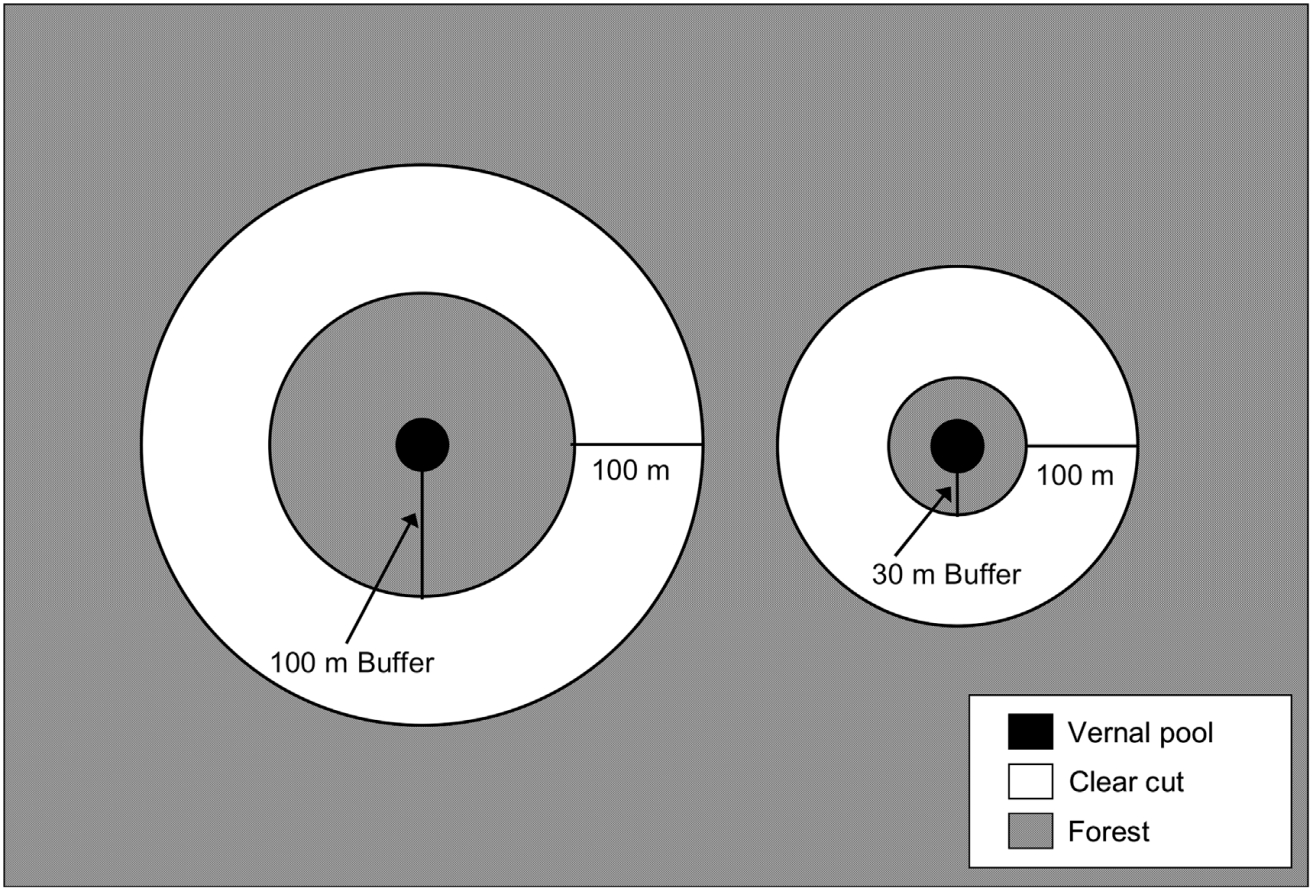
Experimental design implemented at 11 natural ephemeral pools in east-central Maine, USA. Undisturbed buffers of either 100 m (left; n = 4) or 30 m (right; n = 4) were left adjacent to pools and 100-m-wide clearcuts were created around the buffers. Forest beyond the clearcut was undisturbed. No cutting occurred at reference pools (not shown; n = 3).

We randomly assigned each of the remaining 11 pools to one of three treatments: reference (i.e., uncut; N = 3), 100m buffer (N = 4), or 30m buffer (N = 4). From September 2003 to March 2004, the landowner used clearcutting to create experimental buffers at the 100m and 30m treatment pools. The cutting removed all merchantable trees ≥ 5 cm diameter at breast height and slash, though a small quantity of woody debris remained. After cutting, pools in the two buffer treatments had, respectively, a 100-m or 30-m-wide upland buffer encircling the pool and a 100-m-wide concentric clearcut around the buffer (Fig 1). We selected buffer widths typical of those in existing laws, Best Management Practices (i.e., voluntary forestry and development policies designed to protect water and soil quality), and the literature (e.g., [12, 41, 42]).

### Amphibian sampling

During summer and fall 2003, we encircled each of the 11 pools with a drift fence/pitfall trap array [43]. We used plastic silt fencing that was 91 cm tall and buried 8–10 cm deep and positioned fences 5 m upgradient of each pool’s high water mark to minimize flooding risk. We buried pairs of 5.7 liter aluminum cans on opposite sides of the drift fence at 10 m intervals [43]. To prevent amphibian desiccation, we placed a moistened sponge in the bottom of each trap.

From 2004 through 2009, we opened traps in the spring after ice-out and closed traps when a pool was dry for at least seven consecutive days or, in the fall, when amphibians were no longer active due to hard frosts. We uprooted sections of the fence during the winter to allow movement of non-focal species. We checked pitfall traps daily during periods of high amphibian activity (i.e., April-May and July-September) and every one to five days during periods of less amphibian activity (i.e., June and late fall). Due to poor road conditions, we could not access one 30m-buffer pool in 2009. However, our statistical technique was robust to missing data [44], allowing us to use the other five years of data from this pool.

We captured and counted all amphibians entering and exiting the pools and sexed all adult amphibians exiting pools. We assumed all non-gravid adult spotted salamanders and wood frogs leaving a pool had bred in that pool and marked each with a pool-specific toe-clip [45]. After processing each animal, we released it on the opposite side of the fence from the point of capture. From 2005 to 2009, we counted the number of recaptured individuals at each pool. A pool-specific toe-clip was necessary because some pools were in close enough proximity that inter-pool dispersal was possible. However, given the study’s duration and the problems associated with cutting multiple toes [46–48], we did not mark animals separately for each year they were captured or even to indicate the initial capture year. It is thus possible that a few animals were counted more than once in a single year. To minimize the chance of this occurring, we took the following precautions. First, if an individual returned to a pool in the same year that it was first marked (indicated by a fresh toe cut), we only included its initial visit for that year in our analyses. Second, we only counted and marked non-gravid females. Similarly, though closed drift-fence arrays are highly efficient at trapping adult ambystomatids and wood frogs [40, 49, 50], it is possible that some individuals trespassed (i.e., climbed over or under) the fence and were undetected by our counts. To limit potential trespass, we regularly inspected and maintained our fences. We never saw adult wood frogs or spotted salamanders climbing fences during inspections. If trespass did occur, our amphibian counts would be conservative. By contrast, any multiple counts of the same individual in a given year would somewhat inflate our recapture counts. Overall, however, the numbers of animals that trespassed or were counted more than once in a year would be very small compared to the total number of amphibians trapped. Furthermore, we have no reason to expect that multiple-count or trespass rates would differ across treatments or years. Consequently, our analyses should remain valid, despite any multiple counts or trespass.

### Hydroperiod sampling

We calculated hydroperiod for each pool in each year as the number of days a pool held water between ice-out (i.e., < 75% of the pool was covered in ice) and the day the pool dried completely. To facilitate statistical analyses, we assigned a hydroperiod end date of October 28^th^ to pools that did not dry in a given year. We chose this end date because we still had evidence of persistent water in these pools on this date, but it was sufficiently late in the year that most amphibians at our study pools were inactive.

### Statistical analyses

To assess the relative impacts of forestry treatment and hydroperiod on the size and composition of spotted salamander and wood frog active-breeding populations, we developed generalized linear mixed effects regression models using the “glme” function in the correlatedData library of S-Plus 8.0 (Insightful Corporation, Seattle, WA, USA 2007) and the “glmer” function in the lme4 library [51] of R 2.13.0 [52]. We define the active-breeding population as all adults that migrate to a pool and attempt to breed in a given year. Thus, our results apply to a subset of each species’ total local population and do not account for adult salamanders that skip breeding in a given year or juveniles. For the rest of this paper, we refer to our study population simply as “breeding adults.”

We modeled both the sex ratio and proportion of recaptured adults for each species using mixed-effects logistic regression with a logit link. We defined sex ratio, within each study year and at a given pool, as: Number of breeding males / (Number of breeding males + Number of breeding females). We defined the proportion of recaptured adults, within each study year and at a given pool, as: Number of recaptured breeding adults / (Number of recaptured breeding adults + Number of new-captured breeding adults). Note that the number of recaptured breeding adults in a given year is simply a count of the number of marked individuals that returned to breed at a particular wetland in the given year, without distinguishing between adults marked in different years. This definition of recaptured adults derives from our method of marking individuals with a pool-specific toe-clip, but not a year-specific toe-clip. We modeled breeding-adult abundance for each species with mixed-effects Poisson regression, using a log link. We defined breeding-adult abundance as the total number of adults (i.e., recaptured adults + new-captured adults) actively breeding in a given year at a given pool.

We treated year and pool ID as crossed random effects [44] in all models. We modeled the variance-covariance structure for each regression to account for inter-year correlation at individual pools and heterogeneous variance across groups. We used likelihood ratio tests to optimize each regression’s variance-covariance structure. We accounted for intra-pool correlation in the salamander abundance model using a first-order auto-regressive process with year as the time variable. We did not need to define a correlation structure for any other model. We specified the variance structure for all models except the proportion-of-recaptured-salamander model. For salamander sex ratio, we assigned different variances to each study year. For salamander abundance, we allowed the variance to increase exponentially as a function of mean-pool hydroperiod (i.e., the mean hydroperiod for each pool across the six study years). For the proportion-of-recaptured-frogs model, we assigned different variances to each treatment. For frog sex ratio, we allowed the variance to increase, within each study year separately, as a power function of the model’s fitted values. For frog abundance, we allowed the variance to increase, within each treatment separately, as a power function of the standard deviation of pool hydroperiod (as calculated for each pool across the six study years). In their final forms, all models satisfied the assumptions of Poisson or logistic regression.

Our predictor variables were: buffer treatment, mean-pool hydroperiod, standard deviation of pool hydroperiod, an interaction between treatment and mean-pool hydroperiod, and a pair of numeric dummy variables representing an interaction between treatment and study year. We used the first dummy variable (dv.cut) to distinguish whether a pool was subjected to clearcutting or not. We used the second dummy variable (dv.30m) to indicate marginal impacts to the 30m treatment pools. We used dummy variables to represent the effects of study year for several reasons. First, we needed to capture potential changes over time as the clearcuts regenerated. Second, we could not treat year as a categorical variable as this required estimating five coefficients for year alone and our models would not converge with this formulation. When we used the two dummy variables, by contrast, we only needed to estimate two coefficients, we could simultaneously investigate a treatment X year interaction, and our models converged. Because of this formulation, however, we could not test for significant differences between specific years. Rather, we used the estimated dummy-variable coefficients to describe differences in the average rates of change over time across treatments. We used ANOVAs to determine the overall significance of each fixed effect and t/z tests (for S-plus and R models, respectively) to test the significance of the different treatment levels (α = 0.05). We used treatment contrasts to compare the reference treatment to each respective cut treatment (i.e., by default, there was no direct comparison between the 100m and 30m treatments; [44]). We applied a sequential Bonferroni procedure to adjust α-levels during a post hoc comparison between the 100m and 30m treatments for the proportion-of-recaptured-frogs model (i.e., the only model with a significant overall treatment effect, but no significant differences identified by the default inter-treatment comparisons).

Based on our experimental design; prior knowledge that hydroperiod strongly influences both species’ population dynamics; and a desire to approximate natural conditions, where all predictors simultaneously act on the species’ populations, we decided a priori to retain all predictors in each model, whether or not they were statistically significant. We allowed two exceptions to this rule. First, based on an a priori decision, we dropped the hydroperiod interaction from a model when it was not significant and refit the model for the remaining fixed effects. We made this decision principally because, unlike for the other predictors, we had no strong prior reason to think there must be a treatment X hydroperiod interaction. Ultimately, we only included the hydroperiod interaction in the salamander-abundance model. Second, we dropped dv.30m from the proportion-of-recaptured-frogs model because dv.30m and treatment were confounded, with both predictors effectively canceling each other out. For this model alone, we investigated the possibility of confounding factors because raw-data graphs suggested a very strong 30m-treatment effect, which was not supported by the full model results. We tested for confounding factors by systematically dropping each predictor in turn from the model and assessing model fit (using graphs of fitted versus observed values). Ultimately, we retained treatment instead of dv.30m because a reduced model with treatment provided a better fit than one with dv.30m. However, the reduced dv.30m model suggested that the proportion of recaptured frogs in the 30m treatment decreased during the study. We did not test for confounding factors with the other models, as we had no evidence to suggest that this might be a problem for any other model.

### Ethics and data deposition statements

We conducted all of the research in accordance with the rules of the Institutional Animal Care and Use Committee at the University of New Hampshire (IACUC-UNH). IACUC-UNH approved our research protocol, as detailed in permits: 020601 and 050604. None of the captured species were protected or endangered under federal or state law. We conducted the research on private land, with permission from the landowner. For these reasons, no additional permits or permission were needed to conduct this work. The data used in this study are available from the Dryad database (DOI: 10.5061/dryad.547rp).

## Results

**Fig 2:**
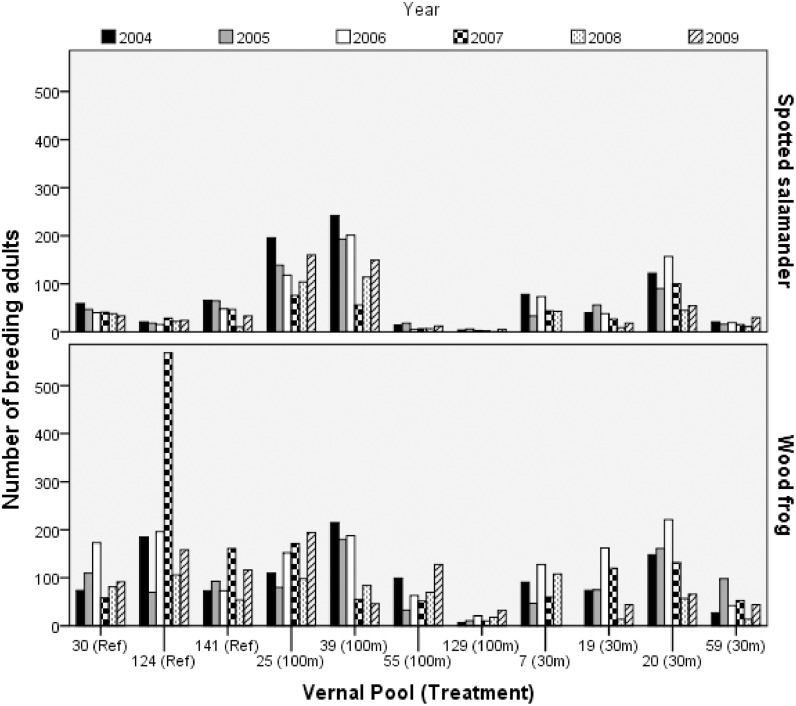
Breeding spotted salamander and wood frog abundance. Shown for populations at 11 natural ephemeral pools in east-central Maine, USA, across the six study years. Each pool is labeled with an identifying number and the applied forestry treatment. Experimental forestry treatments were: reference (uncut), 100m undisturbed buffer, 30m undisturbed buffer.

From 2004 to 2009, we captured 3624 breeding spotted salamanders, including 2811 (78%) new-captures and 812 (22%) recaptures, and 1518 (42%) females and 2099 (58%) males. Similarly, we caught 6521 breeding wood frogs, including 5478 (84%) new-captures, 1014 (16%) recaptures, 2427 (37%) females, and 4072 (63%) males. Breeding-adult abundance, especially for wood frogs, was highly variable within and across pools and years (Table 1, Fig 2). For example, at one reference pool, annual breeding wood frog abundance ranged from 70 to 568, with a mean of 214 frogs, while at a different reference pool, breeding wood frog abundance ranged from 54 to 161, with a mean of 95. Hydroperiod also varied widely in this forest-ephemeral pool ecosystem. One semi-permanent pool never completely dried during the six-year study. By contrast, mean hydroperiod at another pool was about four months, but varied by as much as 49 days.

**Table 1:** Mean and variability of predictor and outcome variables at 11 natural ephemeral pools in east-central Maine, USA. Population parameters represent actively breeding adults only.

	**Mean ± SE**	**Range**		
Mean hydroperiod (days)	125.96 ± 5.98	44.83–197.00^d^		
SD hydroperiod (days)^a^	31.81 ± 1.58	6.32–48.76		
	**Spotted Salamander**		**Wood Frog**	
	**Mean ± SE**	**Range**	**Mean ± SE**	**Range**
**Abundance**				
Reference	36.44 ± 3.95	10–66	135.61 ± 27.58	54–568
100m	76.29 ± 16.46	0–242	88.21 ± 13.35	7–215
30m	49.52 ± 7.96	9–157	86.35 ± 11.18	14–221
**Proportion recaptured** ^b^				
Reference	0.31 ± 0.04	0.06–0.51	0.23 ± 0.04	0.05–0.67
100m	0.23 ± 0.04	0.00–0.63	0.19 ± 0.03	0.03–0.42
30m	0.19 ± 0.03	0.00–0.48	0.09 ± 0.01	0.00–0.18
**Sex ratio** ^c^				
Reference	0.55 ± 0.04	0.10–0.78	0.62 ± 0.04	0.31–0.88
100m	0.59 ± 0.04	0.17–1.00	0.60 ± 0.03	0.14–0.76
30m	0.58 ± 0.03	0.38–0.81	0.62 ± 0.03	0.32–0.86

^a^ Standard deviation of the pool hydroperiod.

^b^ Proportion recaptured = number of recaptured breeding adults / (number of recaptured breeding adults + number of new-captured breeding adults).

^c^ Sex ratio = number of breeding males / (number of breeding males + number of breeding females).

^d^ Some pools did not dry in some years. To facilitate analyses, we assigned such pools a late-fall hydroperiod end date. Mean hydroperiod was calculated using the capped end dates.

### Spotted salamanders

**Fig 3:**
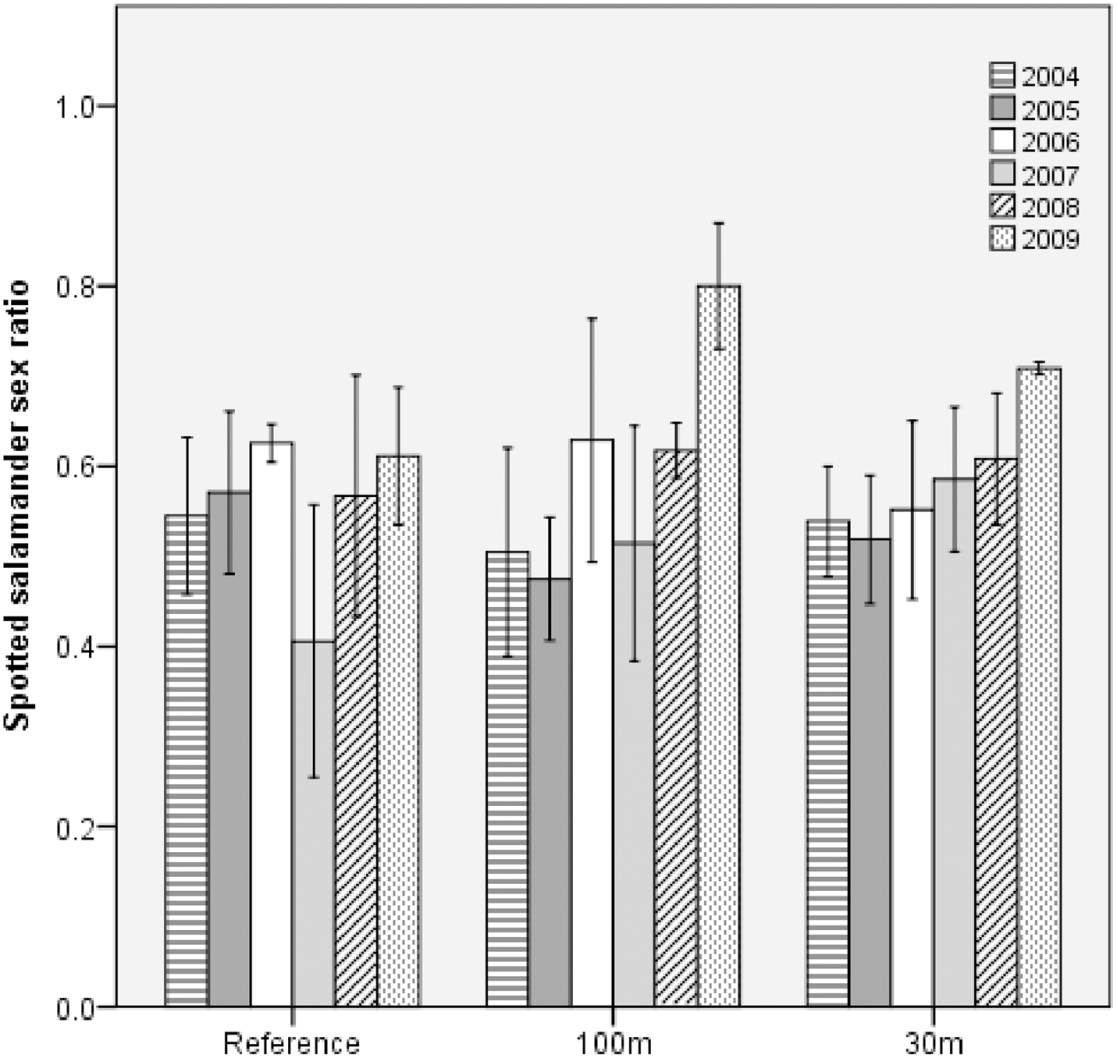
Mean (±1SE) sex ratio of breeding spotted salamanders by forestry treatment and study year. Shown for populations at 11 natural ephemeral pools in east-central Maine, USA. Treatments were: reference (uncut), 100m undisturbed buffer, 30m undisturbed buffer.

Breeding-salamander sex ratio increased significantly in both cut treatments during the study, and a marginally significant trend indicated that the rate of increase in the 30m treatment was slightly lower than that in the 100m treatment (Table 2; Fig 3). For example, at 100m-buffer pools, we predicted approximately equal numbers of each sex in 2004, but 2.3 males per female in 2009. Similarly at 30m-buffer pools, we predicted approximately 1.3 males per female in 2004, but 2 males per female in 2009. At reference pools, by comparison, we predicted 1.1 males per female, on average. In both cut treatments, the male-biased sex ratios were principally driven by a decrease in the number of breeding females.

**Table 2:** Generalized linear mixed effects regression results, showing the relative impact of forestry treatment, hydroperiod, and study year on demographic characteristics of actively breeding spotted salamander and wood frog populations at 11 ephemeral pools in east-central Maine, USA.

Population Metric	Predictor^a^	F value_(df)_	t value_(df)_	Coefficient ± SE
**Spotted Salamander**				
Abundance	treatment(100m)^b^ *mean.hydro^c^	5.65_(2,56)_ *	3.33_(56)_ *	0.03 ± 0.009
	treatment(100m)	4.16_(2,56)_ *	-2.79_(56)_ *	-3.38 ± 1.212
	intercept	12.81_(1,56)_ **	3.58_(56)_ **	3.88 ± 1.084
Proportion Recaptured	treatment(30m)^d^	13.74_(2)_ * ^g^	-2.58* ^g^	-1.27 ± 0.490
	dv.30m^e^	10.12_(1)_ *	3.22*	0.30 ± 0.093
Sex Ratio	dv.cut^f^	28.46_(1,57)_ ***	5.34_(57)_ ***	0.18 ± 0.034
	dv.30m	3.10_(1,57)_ ^•^	-1.76_(57)_ ^•^	-0.09 ± 0.051
**Wood Frog**				
Abundance	mean.hydro	6.17_(1,58)_ *	2.48_(58)_ *	0.01 ± 0.003
	intercept	31.03_(1,58)_ ***	5.57_(58)_ ***	3.35 ± 0.601
Proportion Recaptured	treatment(100m vs 30m)	9.53_(2,48)_ **	4.36_(48)_ **	0.81 ± 0.185
	intercept	8.65_(1,48)_ *	-2.94_(48)_ *	-1.39 ± 0.474
Sex Ratio		ns^h^		

^a^ In general, all models included the following predictors: treatment, mean.hydro, standard deviation of the pool hydroperiod (days), dv.cut, dv.30m, and an interaction between treatment and mean.hydro. Based on an a priori decision, we dropped the interaction from the model when the interaction was not significant. We also dropped dv.30m from the proportion-of-recaptured frog model because this variable was confounded with the main effect of treatment. Only significant fixed-effects results are shown.

^b^ Categorical variable, coded 0 = reference treatment and 1 = 100m treatment.

^c^ Mean.hydro = mean pool hydroperiod in days.

^d^ Categorical variable, coded 0 = reference treatment and 1 = 30m treatment.

^e^ Dv.30m = dummy variable representing the marginal impact of the 30m treatment over the six years of the study.

^f^ Dv.cut = dummy variable representing the difference between the reference treatment and the two cut treatments, over the six years of the study.

^g^ We analyzed the proportion of recaptured salamander model in R 2.13. For this model, we thus used Χ^2^ values to assess overall significance of each variable and z values to compare between individual levels of categorical predictors.

^h^ None of the independent variables were significant predictors of wood frog sex ratio.

*** p < 0.0001;

** p < 0.001;

* p < 0.05;

^•^ 0.05 ≤ p <0.1

**Fig 4:**
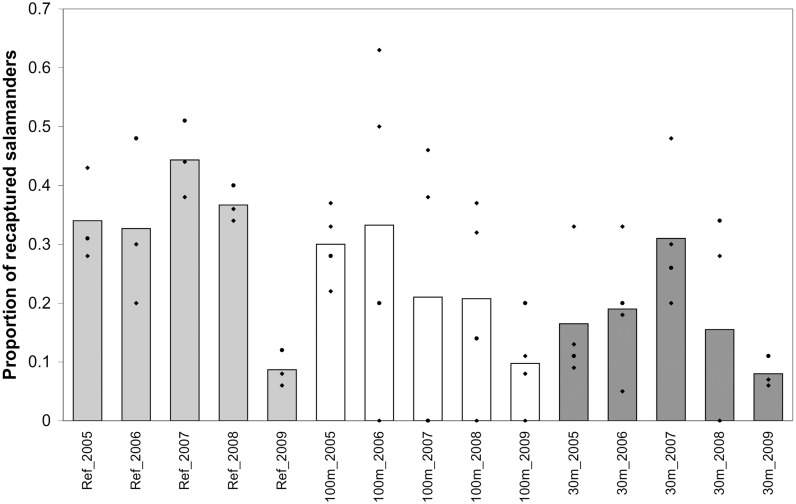
Mean proportion of recaptured breeding spotted salamanders by forestry treatment and study year, with points representing actual values for individual wetlands within each treatment. These data are for populations at 11 natural ephemeral pools in east-central Maine, USA. Treatments were: reference (uncut), 100m undisturbed buffer, 30m undisturbed buffer.

The proportion of recaptured breeding salamanders was significantly lower in the 30m versus reference treatment right after the cut, but significantly increased with time, so that by 2008 (i.e., five years post-cut), recapture proportions at 30m-buffer pools were predicted to be only slightly less than those at reference pools (Fig 4). These trends were largely driven by male and female new-capture abundance, which tended to be higher in the 30m treatment during the first three years of the study, but similar in the two treatments by the study’s end. To a lesser extent, these trends may also be explained by recapture numbers in the 30m treatment, which were low immediately after the cut (but also during the last two study years. In fact, we recaptured no females from the 30m treatment in 2009. However, few salamanders were recaptured in any treatment in 2009 and we could only trap at three of the four 30m-buffer pools in 2009). There was no significant difference in the proportion of recaptured salamanders between the reference and 100m treatments.

**Fig 5:**
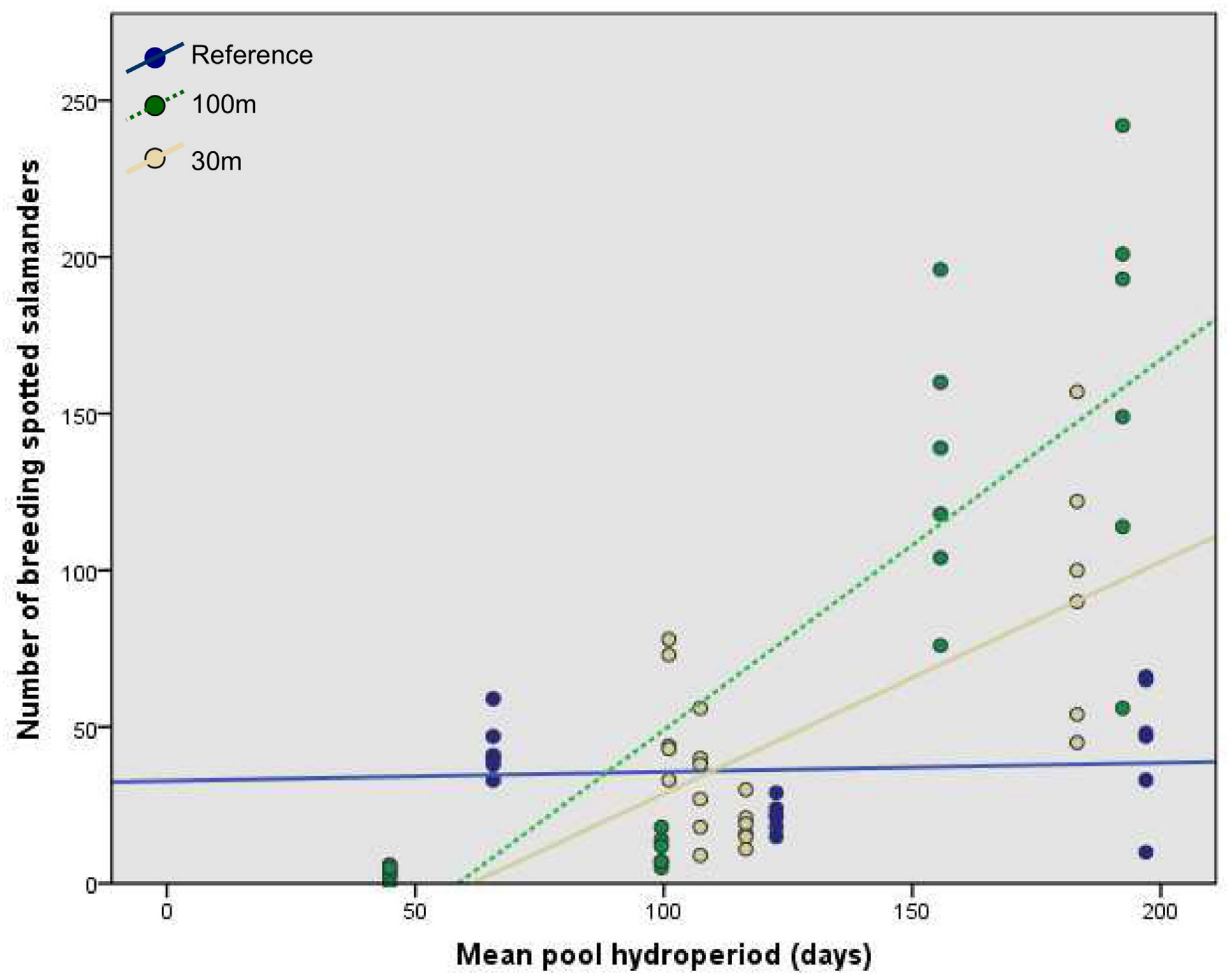
Number of breeding spotted salamanders by experimental forestry treatment and mean pool hydroperiod. Shown for populations at 11 natural ephemeral pools in east-central Maine, USA. Treatments were: reference (uncut), 100m undisturbed buffer, 30m undisturbed buffer.

At short hydroperiod pools, we found significantly fewer breeding spotted salamanders in the 100m treatment than the reference treatment (Fig 5). For instance, if pool mean hydroperiod were 45 days (the minimum mean hydroperiod observed), the number of breeding salamanders at a 100m-buffer pool was predicted to be only about 12% of the abundance at a reference pool. However, abundance increased significantly with mean hydroperiod at the 100m-buffer pools, such that for each additional day a pool held water, the number of breeding adults was predicted to increase by about 3%. We found no significant difference in breeding salamander abundance between the reference and 30m treatments.

### Wood frogs

**Fig 6:**
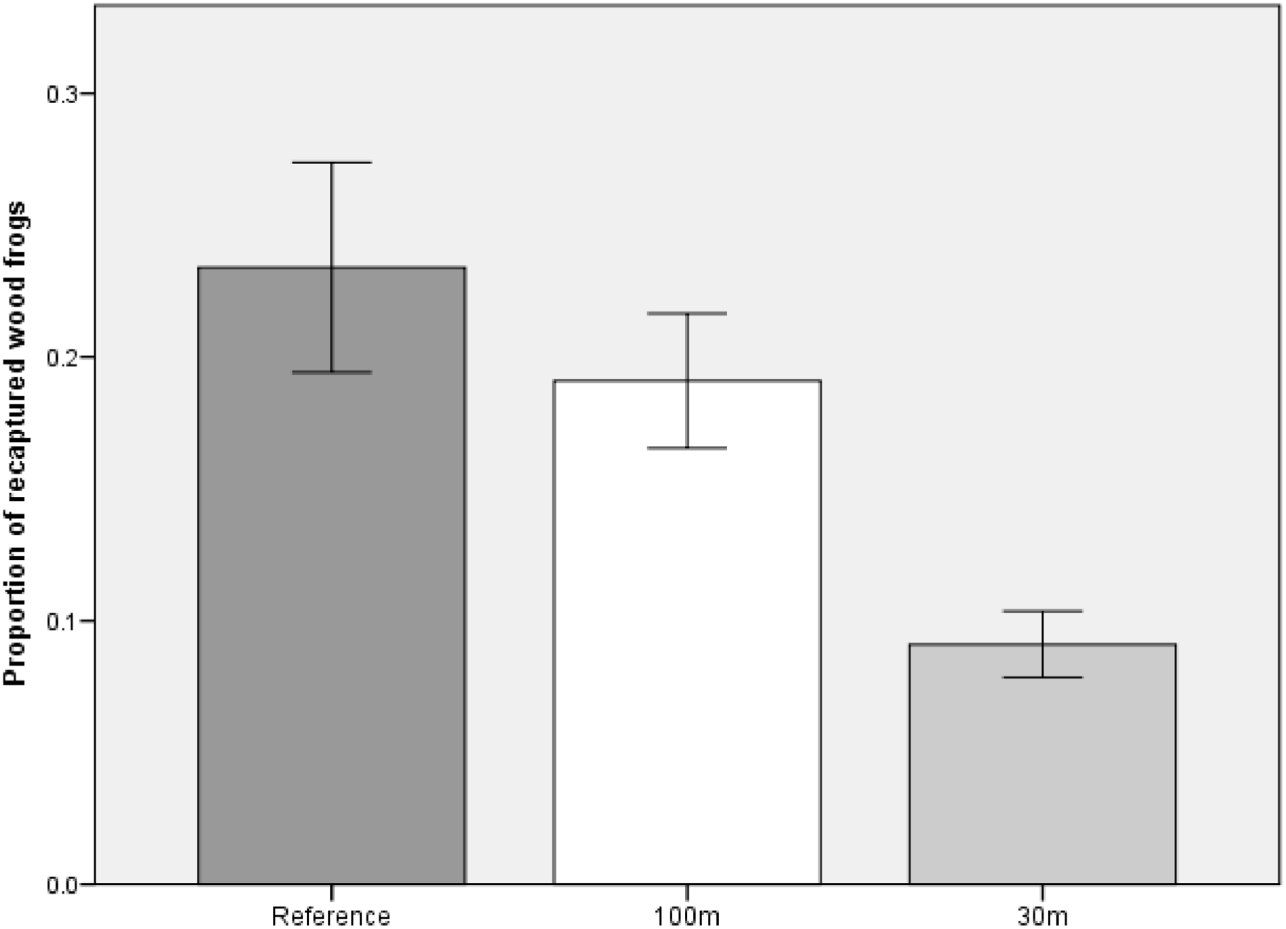
Mean (±1SE) proportion of recaptured breeding wood frogs across three experimental forestry treatments. Shown for populations at 11 ephemeral pools in east-central Maine, USA. Treatments were: reference (uncut); 100m undisturbed buffer; 30m undisturbed buffer.

Though we found no difference in recapture proportion between the reference and either cut treatment, the significant results of the post-hoc test showed that the proportion of recaptured breeding adults in the 30m treatment was predicted to be, on average and for the duration of the study, 62% of that in the 100m treatment (Fig 6). This was largely because both male and female recaptures were scarce in the 30m treatment. In fact, no females were recaptured at 30m-buffer pools in 2008. Buffer treatment was not a significant predictor of breeding wood frog abundance or sex ratio, but we did find significantly more breeding wood frogs at pools with longer hydroperiods. For each additional day a pool held water, abundance was predicted to increase by a factor of 0.7%.

## Discussion

This is the first landscape-scale experiment to explicitly test whether vegetated buffers are an effective tool for managing ephemeral-pool-breeding amphibians and to compare the variable impacts of different buffer widths on breeding-amphibian demography. We found buffer width, time since cut, and pool hydroperiod were all important factors in determining breeding-adult response to clearcutting of terrestrial habitat around ephemeral pools. Contrary to our hypotheses, breeding-population abundance and buffer width were not positively correlated; only one of four negative demographic responses recovered during the six-year study; and wood frogs, where impacted, did not recover faster than spotted salamanders. However, we did find that recaptured adults were least abundant in the narrow (30m) buffer treatment, breeding-salamander sex ratios were skewed in both buffer treatments, and that the effects of buffer width on breeding-salamander abundance were mediated by pool hydroperiod. Our objective with this landscape-scale experiment was to describe breeding-population-level patterns of species response to buffered disturbance. Though we did not test for mechanisms driving these patterns, we explore potential mechanisms in the following discussion.

### Spotted salamanders

#### Sex ratio

The sex ratio of breeding salamanders was significantly altered by buffer treatment. Whereas the sexes were present at reference pools in near equal proportions, males were more than twice as abundant as females in both buffer treatments by the end of the study. Though spotted salamander breeding populations are typically male-biased [33, 53, 54], in our study, sharply biased ratios only occurred at pools where clearcuts disturbed non-breeding habitat. The decline in breeding-female abundance which principally propelled the male-biased sex ratios at the buffer treatments suggests that cutting reduced terrestrial habitat quality, leading females to delay maturity, breed less frequently, experience increased mortality, or switch breeding pools.

Clear cutting can negatively impact habitat quality in multiple ways. Desiccation [55, 56] and predation [57, 58] can be higher, while prey [59–61] and shelter [58, 62] availability can be lower, in clearcut versus intact forest. Salamanders may spend less time foraging in cuts, limiting growth, reproduction, and survival [62, 63]. Clearcuts can also reduce habitat quality through edge effects [64, 65] or if salamanders preferentially occupy remnant forests, causing crowding and resource scarcity [38, 66, 67]. Though spotted salamanders can spend long periods in clearcuts [15], they prefer forest [64, 65] and can evacuate cuts into adjacent forest [68], setting the stage for density-dependent fitness effects. Cuts may disproportionately impact females because females invest more in reproduction and thus have greater energetic needs [69–71]. Females may also be more likely to switch pools because they tend to migrate farther and encounter alternative breeding sites more frequently than males [67].

Increasing a breeding-population’s proportion of males can alter mating behavior, with potentially negative consequences for individual fitness and genetic diversity. Spotted salamanders converge on ephemeral pools to breed each spring [72, 73]. Inter-male competition for females is intense and males physically pursue available females [72]. Increasing the proportion of males can intensify inter-male competition, such that many males fail to mate or contribute their genes to the population [72, 74]. Females exposed to male aggression may experience increased stress, which can translate to impaired immunity and body condition, thereby decreasing future fitness [75, 76]. A shortage of breeding females also constrains potential reproductive output and thus population growth [77, 78].

The male-biased sex ratios and associated effects were likely somewhat stronger in the 100m versus 30m treatment because of differences in how the spatial configuration of each cut treatment overlapped with the sex-biased distribution of salamanders around pools. Within 65m of breeding pools, male spotted salamanders are more common than females, which are more likely to occupy habitat that is farther from pools [38, 39]. At the 100m treatment, where the clearcut occupied the zone between 100 and 200 m from the pools, the negative effects of cutting were likely stronger for females than males, since a greater proportion of males versus females would have remained within the forested buffer, while more females would have ventured into the clearcut. Conversely, at the 30m treatment, where the buffer only extended to 30m and the cut was located between 30 and 130 m from the pools, both males and females were likely impacted by the cut. In support of this reasoning, our data show that in the actively breeding population, predominantly females declined with time in the 100m treatment, whereas both males and females declined over time in the 30m treatment.

#### Recapture proportion

Though we found no effect in the 100m treatment, the proportion of recaptured salamanders in the 30m treatment was reduced just after the cut, but returned nearly to reference levels by the study’s end. An initial influx and subsequent decline of new-captures at 30m-buffer pools largely propelled this trend. New-captures were either immigrants or adults native to a study pool who previously refrained from breeding. The new-capture influx during the first half of the study indicates that more non-breeders or, more likely immigrants, used 30m-buffer pools than reference pools, during the first few years post-cut. We suggest two explanations for this pattern. First, cutting may have increased mortality among established adults or caused them to emigrate to other pools, freeing habitat for immigrants. Second, cutting may have altered salamander orientation or habitat-selection cues, thereby increasing immigrant detection and/or selection of 30m-buffer pools [79]. For instance, it may have been easier for immigrants to detect wind-borne olfactory cues across the narrow buffer and clearcut of the 30m-buffer pools than across a similar distance of forest at the reference pools.

The subsequent drop in new-captures in the 30m treatment, combined with declining recapture numbers,. means the initial influx of new-captures failed to transition to a stable population of actively breeding resident adults and that immigration to 30m-buffer pools decreased. Such patterns suggest habitat quality in and around 30m-buffer pools declined with time. Deteriorating habitat quality could lead to lower growth rates, delaying reproductive maturity [37, 80] or causing adults to skip breeding in some years [81, 82], thus limiting recapture rates among resident adults. Alternatively, reduced habitat quality could lead to lower survival [10, 55, 83] or cause individuals to switch breeding pools [84]. Ultimately, the influx of new-captures did not convert to breeding recaptures, indicating that the 30m treatment likely acted as a sink for numerous adult salamanders.

#### Abundance

Hydroperiod mediated the impact of buffer treatment on breeding-salamander abundance. Compared to reference pools, short-hydroperiod 100m-buffer pools had small breeding populations, while long-hydroperiod 100m-buffer pools had large breeding populations. Short hydroperiods typically support smaller populations [85–87], because fewer metamorphs are produced [88–90] and surviving metamorphs can be small and disease-prone [91, 92]. In resource-rich terrestrial habitats (i.e., at reference pools), such stunted metamorphs may exhibit compensatory or ‘catch-up’ growth, minimizing impacts to population abundance [91, 93, 94]; but this may not be possible in resource-poor terrestrial habitats.

The small breeding populations at short hydroperiod, 100m-buffer pools provide further evidence that clearcutting created a resource-poor terrestrial habitat, whose negative impacts were not fully offset by the 100m buffer. As stated previously, clearcuts can be a harsh environment and cause edge effects and possibly overcrowding in adjacent buffers. These habitat changes could reduce breeding populations if: salamanders emigrate to other breeding pools, experience elevated mortality, delay maturity, or invest less energy in reproduction. In the last case, population fecundity could suffer if females breed less frequently or produce smaller clutches. These negative effects may be masked at long hydroperiod pools where metamorphs are more robust and do not need surplus ‘catch-up’ resources [95, 96].

We cannot easily explain why breeding-salamander abundance at long hydroperiod pools was higher in the 100m versus reference treatment or why we found a hydroperiod effect at 100m, but not 30m, pools. In the former case, breeding abundance was also more variable at 100m-buffer pools, possibly indicating less consistent resource availability in the 100m treatment. In the latter case, we suggest three possible reasons. First, the cut zone in the 100m treatment (i.e., 100–200 m from the pool) may be a more vital source of ‘catch-up’ resources, due to its location or larger area, than the cut zone at 30m-buffer pools (i.e., 30–130 m). Second, resource competition may force stunted metamorphs to settle for sub-optimal, clearcut habitat [66], but because the clearcut is farther from the 100m-buffer pools, migration costs may be higher and metamorphs may have greater energetic deficits upon reaching the cut. Conversely, because of its greater size, salamanders may be less likely to leave the 100m buffer, leading to overcrowding and the associated risks of disease transmission, predation, and malnourishment.

### Wood frogs

#### Recapture proportion

The proportion of recaptured wood frogs was lowest in the 30m treatment, suggesting inferior habitat quality around 30m-buffer pools for at least the first six years post-cut. Habitat switching and/or high mortality of mature individuals could explain the lack of recaptured adults. Wood frogs in the southeastern U.S. are known to change breeding pools in response to habitat disturbance [84]. In a separate study at our research site, multiple radio-tagged wood frogs from 30m-buffer pools migrated through the clearcut to intact forest [16]. Such frogs may have continued moving until they encountered an alternative breeding pool (though the frogs were not tracked long enough to determine if this actually happened). It is also possible that mortality was elevated at the 30m-buffer pools due to both direct and indirect cutting effects (e.g., crushed by a skidder [97, 98], overcrowding in the buffer [99, 100], predation [71]).

Recapture rates were depressed at 30m, but not 100m-buffer, pools. Land out to 130 m from a pool supports large numbers of, and is important winter habitat for, adult wood frogs, but densities decline markedly after 130 m [39, 101]. Thus it is plausible that cutting affected more critical terrestrial habitat for mature frogs at 30m versus 100m-buffer pools. Additionally, adult wood frogs experience negative density dependence when terrestrial habitat is limited [35] and juveniles crowd into suitable habitat, even if it increases mortality [99, 100]. Therefore, it is also possible that overcrowding negatively impacted frog populations, with greater effects at the smaller, 30m buffer where less habitat was available adjacent to pools.

Though recaptured frogs were scarce at 30m-buffer pools, breeding abundance did not differ between treatments, suggesting immigrants crossed cuts to replenish local populations and that local wood frog populations do not critically depend on repeat breeding by mature adults. Radiotracking research confirmed that wood frogs could cross our cuts [16]. Nevertheless, the persistent scarcity of recaptures in the 30m treatment suggests negative habitat impacts, not entirely offset by immigration, that could inflate local extinction probabilities [10]. Though we did not explicitly age study animals, recaptured adults are typically older than new-captured adults; as new-captures are either first-time breeders or immigrants, and most immigrants are relatively young, since dispersal generally occurs during the juvenile phase [102]. Older wood frogs tend to be larger than younger frogs [35, 103]. At our 30m-buffer pools, not only did we recapture fewer frogs, but those we did re-catch were smaller than recaptured reference-treatment frogs [104], suggesting a shift in breeding-population structure towards smaller and/or slower-growing, and possibly less competitive, individuals. In general, small size is associated with reduced competitive ability when mating [103, 105] and the production of smaller eggs [106], less sperm [107], and less competitive larvae [35]. Less competitive larvae are generally smaller at metamorphosis [35, 37], which can feedback to reinforce small adult size and clutch volumes and lead to delayed sexual maturation and increased risk of mortality prior to first reproduction [108, 109]. As a result, fecundity may decline, leaving a population vulnerable to future perturbations [109].

Extinction probability also increases with stochastic variability and permanence of habitat alteration [10]. Our system was highly variable due to inter-annual differences in precipitation and temperature, which jointly drove fluctuating hydroperiods and amphibian productivity. Despite this variability, clearcuts regenerate. We thus expect breeding wood-frog recapture proportions to recover and extinction probabilities to decrease as forests at 30m-buffer pools re-grow. The precise recovery timeframe is unclear, however. In fact, the alternative recaptured-frog model, which included the year X 30m-buffer interaction (i.e., included dv.30m instead of the main treatment effect), suggested a declining proportion of recaptured frogs over our six-year study. Other studies in east-central Maine found reduced wood-frog abundance in clearcuts during the first six years post-cut [110] and reduced landscape permeability among juveniles for 10–20 years post-cut [111]. Our combined results suggest more than six years of regeneration might be needed for clearcuts to develop the moist micro-climates and structured leaf litter necessary to support stable wood-frog breeding populations.

#### Sex ratio and abundance

Breeding wood frog sex ratio and abundance were not significantly impacted by buffer treatment. We were intrigued that sex ratios did not differ between the 30m and reference treatments because previous research suggested females might disproportionately suffer mortality during winter cutting, especially at 30m-buffer pools [39, 40]. By contrast, our results suggest that both sexes experienced similar levels of cutting-induced mortality and/or behavioral changes, allowing for stable relative breeding abundances. Indeed, based on the spatial distribution patterns previously described for wood frogs, we estimate that more than 25% of adults from both sexes could have been directly impacted by winter harvesting of the 30–130 m zone at the 30m-buffer pools [39, 40, 101]. Additionally, radiotracking data from a separate study at our research site indicate modified migratory behavior in the 30m treatment, with each sex adopting a different strategy for seeking viable habitat [16]. While males may have responded to cutting by staying in the buffer, females tended to move quickly through the cut into undisturbed forest [16]. These strategies appear adaptive when compared to reference-pool behavior, where mean emigration distances were 72 ± 28 m and 95 ± 16 m, for males and females, respectively, locating both sexes squarely in what would be the clearcut were they at 30m-buffer pools [16]. Overall, the number of males seeking refuge in the buffer may have been matched by females sheltering in the forest beyond the cut, resulting in similar sex ratios at the 30m and reference treatments.

Breeding-frog abundance, while unaffected by treatment, was correlated with mean-pool hydroperiod. This is consistent with previous work showing that wood frogs are more abundant where ephemeral pools hold water long enough for larvae to develop completely [86, 87, 112]. What distinguishes our study is that hydroperiod was a dominant predictor of breeding wood frog abundance despite the major disturbance of clearcutting. This result reaffirms the importance of metamorph production for breeding wood frog population abundance [35, 37, 113]. Metamorph production is foremost a function of hydroperiod [113, 114] and the most direct link between hydroperiod and adult abundance. In ephemeral-pool ecosystems, hydroperiod, metamorph production, and breeding-adult populations vary widely from year to year [113, 114]. Even without habitat disturbance, wood frog populations have high natural extinction probabilities and depend on boom metamorph production years for persistence [10]. Habitat disturbance likely amplifies this dependence on metamorphs and, ultimately, hydroperiod.

### Landscape context and interspecies comparison

Ultimately, we suggest four major mechanisms to explain the focal species’ negative response to buffer treatments: reduced philopatry, delayed maturity, temporary emigration (i.e., skipped breeding opportunities), and increased mortality. Determining the relative contribution of each mechanism is a ripe area for future research. Nonetheless, we can use landscape context and differences in the species’ life-history strategies to identify the particular mechanisms that might be most important for each species.

Previous research suggests that for both species, population structure depends on the density and configuration of breeding wetlands [84]. Where multiple breeding wetlands are within a few hundred meters of each other and separated by a permeable matrix, adults will switch breeding pools in response to disturbance and the pool-group constitutes the local population [84]. In this scenario, recapture of mature breeders at any single pool would be diminished because adults move among pools. As the inter-pool distance increases beyond the adults’ migratory capacity, inter-pool movement is mostly limited to juvenile dispersal and individual pools likely constitute local populations, supplemented by rescue and recolonization from a larger metapopulation [115]. In this scenario, adults are expected to be quite philopatric to breeding pools and recaptures relatively high. For spotted salamanders and wood frogs, respectively, the maximum definitively recorded adult migration distances are 467 and 394 m [116, 117].

We conducted our study in a relatively moist, working forest in the northeastern U.S. where the forest and wetland habitats that these species prefer are generally abundant. The shortest distance between any of our study pools was 1.4 km, but numerous additional breeding wetlands were present in the landscape. A separate survey of all breeding wetlands, which covered about 50% of our study area and included seven of the 11 study pools, found that the mean nearest neighbor distance between breeding wetlands was 245 and 223 m for spotted salamanders and wood frogs, respectively [118]. At four of the seven surveyed study pools (one each in the 30m and reference treatments and two in the 100m treatment), the nearest breeding wetland was < 550 m away [118, 119], a distance conceivably within the migratory range of adults of both species. Thus, many breeding wetlands in the broader landscape and in our study were likely part of a local population cluster with intermittent adult migration between pools, but multiple other study pools were relatively isolated and likely functioned as independent local populations, with limited inter-pool adult movement. This configuration suggests that for both species, some mature adults could have switched breeding pools in response to the cuts, such that reduced philopatry may partly explain the diminished recapture rates in the 30m treatment. Due to logistical constraints, we did not search for recaptured adults in non-study pools, however, so we cannot confirm this deduction.

Among the other possible mechanisms, examination of each species’ life-history strategy suggests that temporary emigration and increased mortality were also plausible explanations for the salamander results; while greater mortality was a plausible mechanism driving frog-recapture patterns. The spotted salamander is long-lived and iteroparous, with an extended juvenile period [33]. Like other iteroparous species, lifetime reproductive output is maximized if adults breed multiple times during their lifespan; thus, adult survival is key to population persistence [10, 120]. Spotted salamanders normally offset reproductive costs and risks by refraining from breeding in some years, during which their energy and reproductive stores are replenished [81, 82, 121]. Best estimates suggest that about 17% of adults skip breeding in a typical year, but breeding probability is higher for males and increases for both sexes with precipitation, which facilitates migration [73, 122, 123]. It is unlikely that precipitation dictated temporary-emigration patterns in our study, however, since rainfall was relatively high during each breeding season (except one week in March 2004 and three weeks in spring 2006, which were considered abnormally dry, but not droughty, and did not correspond with particularly low recapture rates, abundances, or female captures; [124]).

As explored earlier, cutting likely altered food availability and/or desiccation and predation risks, making it harder for salamanders to build and/or replenish energy and reproductive stores in the cut treatments. Given their plastic reproductive behavior, individual salamanders likely responded to these habitat changes by extending their time to maturity and/or interbreeding interval. If delayed maturation were the principle mechanism, we would expect an up-tick in recapture numbers in the cut treatments in the latter half of the study. Typical maturation times for males and females at these latitudes are three and four years, respectively [121, 125]. If maturation were extended by a couple years, animals born in the first half of the study, who survived and did not disperse, should re-emerge and be recaptured in the second half of the study. That we did not see a recapture up-tick suggests that delayed maturation is not a principle mechanism driving response patterns (though it may play a role, especially if individuals can delay maturation for longer than a couple years).

The recapture patterns are consistent, however, with extended temporary emigration. The maximum observed interbreeding interval for spotted salamanders in an undisturbed population is four years [73]. Using this as a reference, any salamander first caught during or after the second study year, could conceivably wait to breed again until after the study terminated. If salamanders could extend the interbreeding interval even longer, then individuals initially captured during the study’s first year also might not return to breed before the study ended. Thus, relatively few mature breeders would be recaptured in the 30m treatment throughout the study, a pattern relatively consistent with our results. The recapture patterns are also consistent with increased mortality among mature breeders, in that adults who do not survive also cannot be recaptured. If cutting sufficiently degraded habitat conditions, salamanders might experience higher mortality in the 30m versus reference treatment, even if they elected to skip breeding.

When combined with knowledge about the spatial distribution of the sexes, extended temporary emigration and increased mortality are also consistent with the altered sex ratios in both cut treatments and the reduced abundances at short-hydroperiod 100m-treatment pools. Compared to males, female spotted salamanders have a greater reproductive burden, and typically migrate and overwinter farther from breeding pools [38, 39, 101]. Consequently, females were more likely than males to experience negative effects from the cuts, and thus to suffer increased mortality and/or require greater interbreeding intervals. These effects may have increased with time, if habitat conditions in the cut treatments remained bad enough that females were unable to replenish energy stores even with temporary emigration, such that a greater proportion of females died or were unable to breed as the study progressed. Females may have been even more strongly impacted in the 100m versus 30m treatment, because the clearcut in the in 100m treatment was larger in total area, farther from the pool, and thus likely occupied by fewer males than females.

By contrast, wood frogs mature faster and have a shorter life-span and greater clutch size [35, 36]. Though some individuals may breed in up to five years, most breed only once [35]. The wood frog’s principal life-history strategy, then, is to persist through multitudinous offspring, rather than depend on repeat breeding by adults. In line with this strategy, temporary emigration has never been observed for wood frogs, though they can adapt to subpar habitat by delaying maturity [108]. Most males mature as one-year-olds and females as two-year-olds, but age-of-first-reproduction depends on terrestrial resource availability and has been observed to vary by up to two years [35].

It is unlikely that delayed maturation was a principle mechanism driving frog-recapture patterns, however. Similar to salamanders, if delayed maturation were the frogs’ main response to cutting, we would expect an increase in the proportion of recaptured frogs during the study’s second half, when individuals born during the first few study years finally reached maturity. To avoid such an increase, frogs would need to delay maturation through the study’s end, which in some cases would mean for up to five years. It is unlikely that many frogs lived long enough to delay maturity for such a duration, given that: their life-history strategy normally depends on rapid maturation, annual adult survival is only about 20%, and most adults do not live beyond one or two years [35, 37]. Increased mortality among mature breeders, however, could plausibly explain frog-recapture patterns. Breeding uses considerable energy stores and is inherently risky. As a result, low survival rates are typical, even for frogs in undisturbed habitats [35]. If cutting reduced habitat quality, it would be even more difficult for mature frogs to replenish depleted energy stores, leading to increased mortality and precluding recapture.

In drawing conclusions about the primary mechanisms driving species’ responses, we are by extension, making inferences about the effects of buffer treatment on population structure. For the subset of 30m-buffer pools located in close proximity to other breeding wetlands, reduced recapture rates may largely signal inter-pool adult movement, with the population across the pool cluster remaining relatively stable. If we also conclude that mortality explains part of our results, however, while knowing that numerous adults of both species immigrated to the 30m treatment, we must infer that some 30m-buffer pools acted as sink habitat, siphoning adults from the broader (meta)population. This would be a particular problem for salamanders, which rely on adult survival and multiple per capita breeding events for long-term population viability; but perhaps less of a problem for frogs, which typically have lower recapture rates and rely instead on the high productivity of short-lived adults. Finally, temporary emigration implies that salamanders traded present reproductive output for future output [121]. Though this strategy may pay off in the long-term, it leaves local populations, and by extension the broader (meta)population, more vulnerable to short-term perturbations.

### Management implications

This is the first study to explicitly test whether forested buffers are effective for maintaining breeding-adult populations of ephemeral-pool-dependent amphibians in the short-term following terrestrial habitat disturbance [126]. Previous research shows that amphibian abundance generally declines in response to forest cutting [56, 65, 80], even in the relatively moist northeastern U.S. [110]. Our study demonstrates that buffers help mitigate the impacts of clearcutting, but that buffer width plays a critical role in that process.

Spotted-salamander and wood-frog breeding populations responded negatively to clearcutting in the 30m treatment, suggesting 30m buffers may be insufficient for maintaining resilient breeding-populations of both species, at least during the first six years post-disturbance. This was true despite great ecosystem variability, which complicates statistical detection of treatment effects. Though breeding abundance was not directly affected, the sex-ratio and recapture impacts suggest increased vulnerability of 30m breeding-populations to additional stressors (e.g., climate change, disease; [127, 128]). Even if temporary emigration and reduced philopatry temper these ill effects in the long-term or over larger spatial scales, these mechanisms do not negate the fact that breeding populations at individual pools in the 30m treatment had fewer recaptured adults and female salamanders and were thus more vulnerable to perturbations in the short-term than individual pools in the reference treatment. Moreover, some 30m-buffer pools appeared to act as sink habitat for immigrants of both species for the first six years post-cut. With 100m buffers, cutting negatively affected breeding-salamander sex ratio and abundance, but not recapture proportion, suggesting breeding-population processes and habitat quality were less impaired than with 30m buffers. Similarly, no wood-frog metrics were impacted in the 100m treatment. Lagged demographic effects are possible in both cut treatments [10], but unexpected, given forest regeneration. We examined amphibian response to variable buffer sizes, given a static clearcut width, but we recognize that optimal buffer width may vary with clearcut size. Testing other clearcut configurations was beyond our study’s scope, however.

Notwithstanding the major disturbance caused by our clearcuts, hydroperiod was a key driver in this ecosystem. Mean-pool hydroperiod was the only significant predictor of breeding wood-frog abundance. Breeding-salamander abundance was best predicted by an interaction between hydroperiod and buffer treatment. Our results reaffirm that hydroperiod must be factored into these species’ management plans. To maintain viable breeding-populations of both species, land-use planners should prioritize conserving ephemeral pools with medium to long hydroperiods (i.e., > 4 months), a recommendation supported by previous research [85, 86, 112].

Overall, our study provides solid experimental evidence that 30m-wide water-quality buffers, which are assumed to provide wetland-dependent-wildlife habitat, are not sufficient in the short-term to maintain resilient local breeding populations of spotted salamanders and wood frogs, species representative of ephemeral-pool-dependent amphibians in the eastern U.S. Breeding populations in the 100m treatment were relatively more resilient, indicating that 100m buffers may provide adequate habitat in some contexts, especially for wood frogs. Despite the negative impacts from clearcutting that we observed at individual pools, however, inter-pool gene flow was high in this forest for both species [119, 129] and adults at some pools may have adapted to cutting by switching breeding wetlands, suggesting that the broader implications of our results are landscape-dependent. In our landscape, wetlands and forest patches were relatively abundant, precipitation was relatively high, and the clearcut-return interval was several decades [130]. Notwithstanding timber harvest, this landscape was sufficiently permeable to support substantial inter-pool gene flow [119, 129]. However, inter-pool dispersal may be impeded and 100m buffers may inadequately protect local breeding-population resiliency, in landscapes where: cutting is more extensive; water is less plentiful; site preparation reduces habitat quality; the return interval is shorter; or other stressors interact synergistically with forest cutting. This may be especially true in exurban and suburban landscapes where, unlike forestry, development permanently alters habitat suitability [131–133].
